# Association of hepatitis B virus and thyroid hormones during pregnancy

**DOI:** 10.1097/MD.0000000000039925

**Published:** 2024-10-04

**Authors:** Jing Wang, Xiaoqiong Yang, Xiuzhen Liang, Yan Zhang

**Affiliations:** a Department of Clinical Laboratory, Chongqing Red Cross Hospital (People’s Hospital of Jiangbei District), Chongqing, China.

**Keywords:** chronic hepatitis B, genotype, hepatitis B virus, pregnancy, thyroid hormone

## Abstract

This study aims to evaluate the correlation between serum thyroid hormone levels and hepatitis B virus (HBV) DNA and HBV genotypes in pregnant women with chronic hepatitis B. A total of 96 pregnant women with chronic HBV-infected pregnant women between January 2020 and December 2022 were selected as the observational study subjects. About 50 HBV-uninfected pregnant women during the same period were selected as the control group. Serum thyroid hormone levels at different stages of pregnancy, including free triiodothyronine (FT3), free thyroxine (FT4), thyroid stimulating hormone (TSH), thyroperoxidase antibody (TPOAb), and thyroglobulin antibody (TGAb), were compared between the 2 groups. Thyroid hormones levels were also compared between HBV-DNA-positive and HBV-DNA-negative women in the HBV-infected pregnancy group, and to determine the correlation between thyroid hormones levels and HBV-DNA load and HBV genotype in HBV-DNA-positive pregnant women. The TSH levels in mid and late pregnancy and TPOAb levels in early, mid, and late pregnancy of HBV-infected pregnant women were higher than those in the same period in HBV-uninfected pregnant women (*P* < .05). The TPOAb and TGAb levels in early, mid, and late pregnancy of HBV-DNA-positive pregnant women were higher than those in the same period in HBV-DNA-negative pregnant women (*P* < .05). The HBV-DNA load and FT3 or FT4 levels were negatively correlated (*P* < .05), and the HBV-DNA load and TGAb levels were positively correlated (*P* < .05). However, there was no statistical difference in thyroid hormone levels between different HBV genotypes (*P* > .05). The thyroid hormone levels will change in pregnant women infected with hepatitis B virus, and there is a certain correlation between HBV-DNA load and thyroid hormone levels. Therefore, timely monitoring of thyroid hormones and HBV-DNA load can provide early prevention and treatment for HBV infection in pregnant women, ensuring the health of pregnant women and fetuses.

## 1. Introduction

Thyroid hormones perform a variety of fundamental physiological functions and are of great significance for the growth and development of children as well as the normal health of adults. Numerous changes in thyroid function take place during pregnancy, and certain thyroid disorders can have an impact on pregnant women and their fetuses.^[1]^ Sufficient thyroid hormones are crucial for the normal development of the fetus. Moreover, the levels of thyroxine (T4) and triiodothyronine (T3) in the brain are controlled in both space and time to facilitate normal patterns of neuronal migration and differentiation.^[2]^ Thyroid stimulating hormone (TSH) prompts the thyroid gland to generate and release hormones.^[3]^ The fetal thyroid gland is formed between 10 and 12 weeks of pregnancy. It begins to produce thyroid hormones at least from 18 weeks of pregnancy. However, it should be noted that fetal thyroid hormone levels won’t reach those of adults until 36 weeks of pregnancy.^[4–6]^ During the critical period of development in the early pregnancy, the fetus relies on maternal thyroid hormone that crosses the placenta. A deficiency of thyroid hormones during developmental stages can affect the neurocognitive development of children and result in a series of serious adverse consequences later in life.^[3,7]^ Therefore, early detection and targeted treatment of thyroid-related diseases are important for decreasing the risk of pregnancy in women.

HBV infection has a significant impact on global health. The mortality rate of viral hepatitis is higher than that of tuberculosis and human immunodeficiency virus (HIV) infection.^[8]^ It affects over 290 million people globally, causing health problems like hepatitis, cirrhosis, hepatic failure, and hepatocellular carcinoma, leading to 887,000 deaths annually.^[9]^ The economic burden associated with treating these complications is also substantial, placing a strain on healthcare systems worldwide. Over the last ten years, epidemiology has changed due to vaccination, screening programs, and population movement. Astonishingly, the predominant mode of HBV infection is mother-to-child transmission (MTCT) currently, making up approximately 50% of patients globally.^[10]^ In regions with a high prevalence of the disease, MTCT is the primary route of infection, and the risk of developing chronicity is significant. Nevertheless, although MTCT of HBV is the main transmission route in many areas around the world, research on the natural progression of the infection during pregnancy is relatively scarce. Efforts should be made to enhance prevention and treatment strategies to protect both mothers and infants from HBV infection and reduce its global impact.

Moreover, the liver serves as a target organ for thyroid hormones and holds an important role in the metabolism of thyroid hormones.^[11]^ When HBV invades and damages the liver, it causes dysfunction of the thyroid gland and leads to abnormal changes in thyroid hormone levels. Researches have shown that thyroid disorders are connected with HBV-related liver cirrhosis or acute-on-chronic liver failure (ACLF).^[12,13]^ However, due to the special physiological state of pregnancy, whether thyroid hormone secretion in pregnant women with hepatitis B is different from that in pregnant women without hepatitis B remains unclear. Currently, there are few studies on thyroid hormone secretion in pregnant women with hepatitis B. Therefore, this study aimed to investigate the relationship between thyroid hormone levels (including FT3, FT4, TSH, TPOAb, and TGAb) and HBV.

## 2. Material and methods

### 2.1. General data

From January 2020 to December 2022, we enrolled 96 pregnant women with chronic HBV infection who were to give birth for this study. Inclusion criteria were as follows: patients meeting the diagnostic criteria for chronic HBV carriers in the Guidelines for the Prevention and Treatment of Hepatitis B Infection (2015 Edition); patients with single pregnancy; patients without liver cirrhosis, no obvious thyroid enlargement, no history of thyroid disease and other pregnancy complications. Average age (28.9 ± 4.8) years old. The exclusion criteria were as follows: body mass index (BMI) > 30 kg/m^2^; Infection with hepatitis A, C, or E virus; other pathogenic infections; patients with hepatic injury complicated by the administration of antitumor drugs; and a history of diabetes mellitus or congenital heart disease. Simultaneously, 50 HBV-uninfected pregnant women were included in the control group in the same period. Average age (28.3 ± 4.7) years old. There was no statistically significant difference in the age of HBV-infected pregnant and control groups (*P* > .05).

All subjects were informed about the study’s content and participated by signing their consent forms voluntarily. This study was approved by the Ethics Committee of the hospital before implementation. The stages of pregnancy are as follows: T1 period is early pregnancy (pregnancy ≤ 12 weeks), T2 period is mid pregnancy (13th to 27th week of pregnancy), and T3 period is late pregnancy (pregnancy 28 weeks to delivery).

### 2.2. Methods

#### 2.2.1. Thyroid hormone level detection

Fasting venous blood was collected from pregnant women and centrifuged at 3500 rpm. The separated serum was used to detect FT3, FT4, TSH, TPOAb, and TGAb using a fully automated chemiluminescence immunoassay analyzer (Unicel Dxl 800).

#### 2.2.2. HBV-DNA and genotype detection

The separated serum of HBV-infected pregnant women was used to detect HBV-DNA quantification and HBV genotyping through qRT-PCR using the SLAN-96P PCR apparatus, with the kit provided by Hunan Shengxiang Bio-tech Co., Ltd.

### 2.3. Statistics

SPSS software (version 20.0) was used for the data analysis. Measurement data were presented as means ± standard deviation, and the *t*-test was used for comparison. For count data, the chi-square test was employed for analysis. Pearson correlation analysis was utilized to determine the correlation between thyroid hormone levels and DNA load. *P* < .05 was regarded as statistically significant.

## 3. Results

### 3.1. Comparison of thyroid hormone levels between HBV-infected and uninfected pregnant women

The TSH levels in the mid and late pregnancy (1.90 ± 0.79, 2.47 ± 0.92 µIU/mL) of HBV-infected pregnancy group were higher than those in HBV-uninfected pregnancy group during the same period (1.60 ± 0.79, 2.07 ± 0.91 µIU/mL, *P < *.05). TPOAb levels in the early, mid, and late pregnancy (9.07 ± 26.01, 11.19 ± 30.43, 9.87 ± 24.00 IU/mL) of HBV-infected pregnancy group were higher than those in HBV-uninfected pregnancy group during the same period (4.22 ± 10.43, 5.45 ± 12.82, 5.40 ± 10.64 IU/mL, *P < *.05). There were no significant differences in FT3, FT4, and TGAb levels between the HBV-infected pregnancy group and the control group at different gestational stages (*P > *.05) (Table 1).

**Table 1:** Comparison of Thyroid hormone levels between HBV-infected pregnancy group and control group.

Group	Pregnancy period	FT3 (pmol/L)	FT4 (pmol/L)	TSH (µIU/mL)	TPOAb (IU/mL)	TGAb (IU/mL)
HBV-infected pregnancy group (n = 96)	T1	5.23 ± 0.72	10.84 ± 2.43	1.32 ± 1.11	9.07 ± 26.01*	5.54 ± 16.15
	T2	4.82 ± 0.52	10.55 ± 3.55	1.90 ± 0.79*	11.19 ± 30.43*	5.30 ± 14.82
	T3	4.48 ± 0.50	8.25 ± 1.58	2.47 ± 0.92*	9.87 ± 24.00*	5.66 ± 13.52
Control group (n = 50)	T1	5.39 ± 0.69	10.87 ± 1.67	1.28 ± 0.70	4.22 ± 10.43	3.14 ± 8.98
	T2	4.92 ± 0.51	9.99 ± 1.46	1.60 ± 0.79	5.45 ± 12.82	3.21 ± 7.36
	T3	4.56 ± 0.55	8.51 ± 1.31	2.07 ± 0.91	5.40 ± 10.64	4.30 ± 10.73

HBV = hepatitis B virus, FT3 = free triiodothyronine, FT4 = free thyroxine, TSH = thyroid stimulating hormone, TPOAb = thyroperoxidase, TGAb = thyroglobulin antibody.

* Compared to same pregnancy period with the control group, *P < *.05.

### 3.2. Comparison of thyroid hormone levels between HBV-DNA positive and HBV-DNA negative pregnant women with HBV infection

Among the 96 pregnant women infected with HBV, there were 34 HBV-DNA-positive pregnant women and 62 HBV-DNA-negative pregnant women. The TPOAb and TGAb levels in the early, mid, and late pregnancy of HBV-DNA-positive group were higher than those during the same period of HBV-DNA-negative group (*P < *.05). There were no significant differences in FT3, FT4 and TSH levels between the HBV-DNA-positive group and HBV-DNA-negative group at different gestational stages (*P* > .05) (Table 2).

**Table 2:** Comparison of thyroid hormone levels between HBV-DNA-positive and HBV-DNA-negative pregnant women with HBV infection.

Group	Pregnancy period	FT3 (pmol/L)	FT4 (pmol/L)	TSH (µIU/mL)	TPOAb (IU/mL)	TGAb (IU/mL)
HBV-DNA positive group (n = 34)	T1	5.37 ± 0.77	10.76 ± 2.86	1.45 ± 1.08	14.95 ± 32.21*	10.84 ± 21.34*
	T2	4.84 ± 0.51	11.05 ± 5.68	2.02 ± 0.75	18.49 ± 37.60*	10.91 ± 21.74*
	T3	4.55 ± 0.49	7.87 ± 1.47	2.50 ± 1.03	15.76 ± 29.04*	9.88 ± 16.85*
HBV-DNA negative group (n = 62)	T1	5.16 ± 0.68	10.89 ± 2.18	1.25 ± 0.98	5.84 ± 22.28	2.63 ± 11.64
	T2	4.82 ± 0.53	10.28 ± 1.39	1.84 ± 0.81	7.19 ± 25.13	2.22 ± 7.71
	T3	4.44 ± 0.50	8.45 ± 1.61	2.45 ± 0.87	6.64 ± 20.27	3.35 ± 10.76

HBV = hepatitis B virus, FT3 = free triiodothyronine, FT4 = free thyroxine, TSH = thyroid stimulating hormone, TPOAb = thyroperoxidase, TGAb = thyroglobulin antibody.

* Compared to same pregnancy period with the HBV-DNA-negative group, *P < *.05.

### 3.3. Correlation analysis of HBV-DNA load and thyroid hormone levels

Comparing the HBV-DNA load during different pregnancy periods, the HBV-DNA load in late pregnancy (4.42 ± 0.72 lgcopies/mL) was higher than those in early and mid pregnancy (3.78 ± 0.87, 3.95 ± 0.54 lgcopies/mL, *P < *.05) (Table 3).

**Table 3:** Comparison of HBV-DNA load at different gestational stages.

Pregnancy period	HBV-DNA (lgcopies/mL)
T1	3.78 ± 0.87
T2	3.95 ± 0.54
T3	4.42 ± 0.72*†
*F*	8.864
*P*	0.001

* Compared to T1 group, *P < *.05.

† Compared to T2 group, *P < *.05.

Pearson correlation analysis was used to determine the correlation between thyroid hormone levels and HBV-DNA load. HBV-DNA load is negatively correlated with FT3 or FT4 levels (*r* = −0.298, −0.268, *P < *.05). HBV-DNA load is positively correlated with TGAb levels (*r* = 0.269, *P < *.05). There was no significant correlation between HBV-DNA load and TSH or TPOAb levels (*P* > .05) (Table 4).

**Table 4:** Correlation coefficient of HBV-DNA load and thyroid hormone levels (*R* value).

Variable	FT3	FT4	TSH	TPOAb	TGAb
HBV-DNA load	−0.298*	−0.268*	0.168	0.147	0.269*

HBV = hepatitis B virus, FT3 = free triiodothyronine, FT4 = free thyroxine, TSH = thyroid stimulating hormone, TPOAb = thyroperoxidase, TGAb = thyroglobulin antibody.

* *P<*0.05.

### 3.4. Comparison of thyroid hormone levels among different HBV genotypes

Among the 34 HBV-DNA-positive pregnant women, there were 22 cases of genotype B and 12 cases of genotype C, and no other genotypes were found. However, there was no significant difference in FT3, FT4, TSH, TPOAb, and TGAb levels between the 2 groups (*P* > .05) (Table 5).

**Table 5:** Comparison of thyroid hormone levels levels among different HBV genotypes.

Group	Pregnancy period	FT3 (pmol/L)	FT4 (pmol/L)	TSH (µIU/mL)	TPOAb (IU/mL)	TGAb (IU/mL)
Genotype B (n = 22)	T1	5.26 ± 0.60	10.87 ± 3.04	1.49 ± 1.01	14.37 ± 31.24	8.62 ± 17.98
	T2	4.88 ± 0.54	11.33 ± 6.89	2.08 ± 0.86	18.06 ± 39.38	9.94 ± 22.33
	T3	4.52 ± 0.53	7.92 ± 1.56	2.62 ± 1.14	14.26 ± 28.67	8.20 ± 15.86
Genotype C (n = 12)	T1	5.57 ± 1.02	10.55 ± 2.62	1.37 ± 1.26	16.02 ± 32.52	14.93 ± 26.86
	T2	4.77 ± 0.46	10.55 ± 2.36	1.93 ± 0.86	19.29 ± 35.78	12.69 ± 21.44
	T3	4.62 ± 0.42	7.80 ± 1.37	2.36 ± 0.77	18.51 ± 30.79	12.97 ± 18.86

HBV = hepatitis B virus, FT3 = free triiodothyronine, FT4 = free thyroxine, TSH = thyroid stimulating hormone, TPOAb = thyroperoxidase, TGAb = thyroglobulin antibody.

## 4. Discussion

Thyroid disease is a common endocrine disorder, and women of childbearing age are high-risk population for thyroid diseases. During pregnancy, the placenta secretes a large amount of hormones, Pregnant women’s hypothalamic-pituitary-thyroid axis system is in a special state of stress, and the maternal immune status also undergo changes. Therefore, pregnancy has a certain impact on the production and metabolism of thyroid hormones, and pregnant women are prone to hypothyroidism. Hypothyroidism during pregnancy can increase the occurrence rate of poor pregnancy prognosis, while potentially increasing the risk of neurocognitive impairment in offspring potentially.^[14–17]^ Therefore, strengthening the detection of thyroid function during pregnancy will be beneficial for treating the corresponding disease, as well as effectively preventing and reducing thyroid dysfunction in pregnant women. Studies have shown that gestational serum FT4 and FT3 decrease with the increase of gestational weeks while TSH increases. The prevalence rate of subclinical hypothyroidism increases with gestational stages, particularly in late pregnancy.^[18,19]^

Thyroid peroxidase (TPO) plays a central role in thyroid hormone synthesis, and serves as a major autoantigen in autoimmune thyroid disorders. Thyroglobulin (TG) is produced by thyroid follicular cells and it plays a crucial role in thyroid hormone synthesis.^[20–22]^ Elevated levels of TPOAb and TGAb indicate thyroid inflammation, and these antibodies can be detected in 10 to 20 percent of women at childbearing age.^[23,24]^ Approximately one-third of women with subclinical hypothyroidism have antithyroid peroxidase antibodies that can be detected, and positive TPOAb and/or TGAb are considered significant risk factors for hypothyroidism during pregnancy and in the postpartum period.^[25,26]^ TPOAb and TGAb are closely related to various diseases during pregnancy. Research has found that TPOAb is positive in pregnancy, even if the thyroid function is normal, it can still cause fetal intellectual development deficiency, so emphasizing the testing of TPOAb and TGAb during pregnancy.^[27–29]^

In both health and disease, there exists a complex connection between the thyroid and the liver. The liver has an essential physiological function in the activation and inactivation, transportation, and metabolism of thyroid hormones. On the contrary, thyroid hormones influence the activities and hepatic metabolism of hepatocytes.^[11,30]^ It has been reported that there is thyroid dysfunction in severe liver diseases. Hepatitis B virus infection can cause thyroid dysfunction, and TSH was determined to have the greatest potential as a prognostic predictor for hepatitis B virus-related ACLF among thyroid hormones.^[31,32]^ This study shows that TSH levels in the mid and late pregnancy of HBV-infected pregnant women were higher than the same period in HBV-uninfected pregnant women (*P < *.05). TPOAb levels in the early, mid, and late pregnancy of HBV-infected pregnant women were higher than those in the same period in HBV-uninfected pregnant women (*P < *.05). Moreover, TPOAb and TGAb levels in the early, mid, and late pregnancy of HBV-DNA positive pregnant women were higher than those in the same period in HBV-DNA-negative pregnant women (*P < *.05). By comparing the HBV-DNA load of different gestational stages, it was found that the HBV-DNA load increases with the gestational stage. HBV-DNA load is negatively correlated with FT3 or FT4 levels and positively correlated with TGAb levels (*P < *.05).

HBV genotypes have specific geographical distributions. Genotype A is common in Western Europe countries such as Sweden, North American countries such as the United States, and some regions of Africa such as South Africa. Genotypes B and C are widespread in East Asia countries such as China and Southeast Asia. Genotype D is predominant Mediterranean region, the Middle East, and parts of South Asia. Genotype F is discovered in South America and some regions of Central America.^[33]^ The HBV genotype is closely related to the severity of the disease, clinical outcomes, and antiviral treatment response.^[34]^ Among 34 cases of HBV-DNA-positive pregnant women in our study, 22 cases of genotype B and 12 cases of genotype C were detected, but there was no statistically significant difference in the comparison of FT3, FT4, TSH, TPOAb, and TGAb levels between the 2 groups.

In summary, the thyroid hormone levels will change in pregnant women infected with HBV, and there is a certain correlation between HBV-DNA load and thyroid hormone levels. High DNA load may increase the risk of thyroiditis or hypothyroidism. Therefore, timely monitoring of thyroid hormones and HBV-DNA load can provide early prevention and treatment for HBV infection in pregnant women, ensuring the health of pregnant women and fetuses. However, the sample size of this study is limited, and we have not tracked the outcomes of pregnant women. In the future, larger sample studies are needed on pregnant women infected with HBV to elucidate the clinical outcomes and mechanisms of thyroid hormones and HBV infection during pregnancy.

## Author contributions

**Conceptualization:** Jing Wang.

**Data curation:** Xiaoqiong Yang.

**Methodology:** Jing Wang.

**Supervision:** Yan Zhang.

**Writing – original draft:** Jing Wang.

**Writing – review & editing:** Yan Zhang, Xiuzhen Liang.

## References

[1] Koibuchi N. The role of thyroid hormone on cerebellar development. Cerebellum. 2008;7:530–3. 18923818 10.1007/s12311-008-0069-1

[2] Prezioso G Giannini C Chiarelli F. Effect of thyroid hormones on neurons and neurodevelopment. Horm Res Paediatr. 2018;90:73–81. 30157487 10.1159/000492129

[3] Krassas GE Poppe K Glinoer D. Thyroid function and human reproductive health. Endocr Rev. 2010;31:702–55. 20573783 10.1210/er.2009-0041

[4] Shepard TH. Onset of function in the human fetal thyroid: biochemical and radioautographic studies from organ culture. J Clin Endocrinol Metab. 1967;27:945–58. 5339119 10.1210/jcem-27-7-945

[5] Burrow GN Fisher DA Larsen PR. Maternal and fetal thyroid function. N Engl J Med. 1994;331:1072–8. 8090169 10.1056/NEJM199410203311608

[6] Thorpe-Beeston JG Nicolaides KH Felton CV Butler J McGregor AM. Maturation of the secretion of thyroid hormone and thyroid-stimulating hormone in the fetus. N Engl J Med. 1991;324:532–6. 1899469 10.1056/NEJM199102213240805

[7] Bernal J. Thyroid hormone receptors in brain development and function. Nat Clin Pract Endocrinol Metab. 2007;3:249–59. 17315033 10.1038/ncpendmet0424

[8] Stanaway JD Flaxman AD Naghavi M. The global burden of viral hepatitis from 1990 to 2013: findings from the Global Burden of Disease Study 2013. Lancet. 2016;388:1081–8. 27394647 10.1016/S0140-6736(16)30579-7 PMC5100695

[9] Nguyen MH Wong G Gane E Kao JH Dusheiko G. Hepatitis B virus: advances in prevention, diagnosis, and therapy. Clin Microbiol Rev. 2020;33:e00046–19. 32102898 10.1128/CMR.00046-19 PMC7048015

[10] Rybicka M Bielawski KP. Recent advances in understanding, diagnosing, and treating hepatitis B virus infection. Microorganisms. 2020;8:1416. 32942584 10.3390/microorganisms8091416 PMC7565763

[11] Malik R Hodgson H. The relationship between the thyroid gland and the liver. QJM. 2002;95:559–69. 12205333 10.1093/qjmed/95.9.559

[12] Tas A Koklu S Beyazit Y. Thyroid hormone levels predict mortality in intensive care patients with cirrhosis. Am J Med Sci. 2012;344:175–9. 22143128 10.1097/MAJ.0b013e318239a666

[13] Chen JF Weng WZ Huang M. The impact of serum thyroid-stimulation hormone levels on the outcome of hepatitis B virus related acute-on-chronic liver failure: an observational study. BMC Gastroenterol. 2022;22:330. 35799116 10.1186/s12876-022-02406-7 PMC9260984

[14] Batistuzzo A Ribeiro MO. Clinical and subclinical maternal hypothyroidism and their effects on neurodevelopment, behavior and cognition. Arch Endocrinol Metab. 2020;64:89–95. 32187263 10.20945/2359-3997000000201 PMC10522279

[15] Zhao W Li X Xia X Gao Z Han C. Iodine nutrition during pregnancy: past, present, and future. Biol Trace Elem Res. 2019;188:196–207. 30218312 10.1007/s12011-018-1502-z

[16] Thompson W Russell G Baragwanath G Matthews J Vaidya B Thompson-Coon J. Maternal thyroid hormone insufficiency during pregnancy and risk of neurodevelopmental disorders in offspring: a systematic review and meta-analysis. Clin Endocrinol (Oxf). 2018;88:575–84. 29325223 10.1111/cen.13550 PMC5888183

[17] Kiran Z Sheikh A Malik S. Maternal characteristics and outcomes affected by hypothyroidism during pregnancy (maternal hypothyroidism on pregnancy outcomes, MHPO-1). BMC Pregnancy Childbirth. 2019;19:476. 31805890 10.1186/s12884-019-2596-9 PMC6896307

[18] Yu Y Li X Jiang S. Serum thyroid hormone levels among Chinese pregnant women. Gynecol Endocrinol. 2017;33:774–8. 28447527 10.1080/09513590.2017.1320375

[19] Geno KA Nerenz RD. Evaluating thyroid function in pregnant women. Crit Rev Clin Lab Sci. 2022;59:460–79. 35293284 10.1080/10408363.2022.2050182

[20] Taurog A. Molecular evolution of thyroid peroxidase. Biochimie. 1999;81:557–62. 10403190 10.1016/s0300-9084(99)80110-2

[21] Carayanniotis G. The cryptic self in thyroid autoimmunity: the paradigm of thyroglobulin. Autoimmunity. 2003;36:423–8. 14669951 10.1080/08916930310001602975

[22] Ruf J Carayon P. Structural and functional aspects of thyroid peroxidase. Arch Biochem Biophys. 2006;445:269–77. 16098474 10.1016/j.abb.2005.06.023

[23] Prummel MF Wiersinga WM. Thyroid peroxidase autoantibodies in euthyroid subjects. Best Pract Res Clin Endocrinol Metab. 2005;19:1–15. 15826919 10.1016/j.beem.2004.11.003

[24] Balucan FS Morshed SA Davies TF. Thyroid autoantibodies in pregnancy: their role, regulation and clinical relevance. J Thyroid Res. 2013;2013:182472. 23691429 10.1155/2013/182472 PMC3652173

[25] Casey BM Dashe JS Wells CE McIntire DD Leveno KJ Cunningham FG. Subclinical hyperthyroidism and pregnancy outcomes. Obstet Gynecol. 2006;107(2 Pt 1):337–41. 16449121 10.1097/01.AOG.0000197991.64246.9a

[26] Korevaar TI de Rijke YB Chaker L. Stimulation of thyroid function by human chorionic gonadotropin during pregnancy: a risk factor for thyroid disease and a mechanism for known risk factors. Thyroid. 2017;27:440–50. 28049387 10.1089/thy.2016.0527

[27] Zhang Q Wang Z Sun M. Association of high vitamin d status with low circulating thyroid-stimulating hormone independent of thyroid hormone levels in middle-aged and elderly males. Int J Endocrinol. 2014;2014:631819. 24693286 10.1155/2014/631819 PMC3947886

[28] Yuan N Sun J Li Z Chai S Zhang X Ji L. Relationship between anti-thyroid peroxidase antibody positivity and pregnancy-related and fetal outcomes in Euthyroid women: a single-center cohort study. BMC Pregnancy Childbirth. 2020;20:491. 32847542 10.1186/s12884-020-03176-4 PMC7449005

[29] Deng M Zhong X Jiang W Wang Y Dong X. Clinical analysis of chronic lymphocytic thyroiditis in gestational period and pregnancy outcome. J Chongqing Med Univ. 2021;46:411–6.

[30] Piantanida E Ippolito S Gallo D. The interplay between thyroid and liver: implications for clinical practice. J Endocrinol Invest. 2020;43:885–99. 32166702 10.1007/s40618-020-01208-6

[31] Tu Y Ji F Yang J. Weighted thyroid-stimulating hormone disturbance in prognosis of hepatitis B virus-related acute-on-chronic liver failure. Hepatol Res. 2024;54:151–61. 37768830 10.1111/hepr.13970

[32] Chawalitmongkol K Maneenil K Thungthong P Deerochanawong C. Prevalence and associated factors for thyroid dysfunction among patients on targeted therapy for cancers: a single-center study from Thailand. J ASEAN Fed Endocr Soc. 2023;38:77–85. 38045662 10.15605/jafes.038.02.18 PMC10692429

[33] Allain JP. Epidemiology of Hepatitis B virus and genotype. J Clin Virol. 2006;36(Suppl 1):S12–17. 16831687 10.1016/s1386-6532(06)80003-x

[34] Chen J Li L Yin Q Shen T. A review of epidemiology and clinical relevance of Hepatitis B virus genotypes and subgenotypes. Clin Res Hepatol Gastroenterol. 2023;47:102180. 37479136 10.1016/j.clinre.2023.102180

